# Behavioral and Amygdala Biochemical Damage Induced by Alternating Mild Stress and Ethanol Intoxication in Adolescent Rats: Reversal by Argan Oil Treatment?

**DOI:** 10.3390/ijms251910529

**Published:** 2024-09-30

**Authors:** Hicham El Mostafi, Aboubaker Elhessni, Hanane Doumar, Tarik Touil, Abdelhalem Mesfioui

**Affiliations:** 1 Laboratory of Biology and Health, Department of Biology, Faculty of Sciences, Ibn Tofail University, Kenitra 14 000, Morocco; elhessni70@yahoo.fr (A.E.); doumarhanan89@gmail.com (H.D.); a.mesfioui@yahoo.fr (A.M.); 2 Higher Institute of Nursing and Health Professions of Rabat, Rabat 4502, Morocco; ttouil@gmail.com

**Keywords:** adolescence, ethanol binge-like, mild stress, amygdala damage, Argan oil, neuroprotection, Wistar rat

## Abstract

Adolescence is a critical period when the effects of ethanol and stress exposure are particularly pronounced. Argan oil (AO), a natural vegetable oil known for its diverse pharmacological benefits, was investigated for its potential to mitigate addictive-like behaviors and brain damage induced by adolescent intermittent ethanol intoxication (IEI) and unpredictable mild stress (UMS). From P30 to P43, IEI rats received a daily ip ethanol (3 g/kg) on a two-day on/two-day off schedule. On alternate days, the rats were submitted to UMS protocol. Next, a two-bottle free access paradigm was performed over 10 weeks to assess intermittent 20% ethanol voluntary consumption. During the same period, the rats were gavaged daily with AO (15 mL/kg). Our results show that IEI/UMS significantly increased voluntary alcohol consumption (from 3.9 g/kg/24 h to 5.8 g/kg/24 h) and exacerbated withdrawal signs and relapse-like drinking in adulthood. Although AO treatment slightly reduced ethanol intake, it notably alleviated withdrawal signs during abstinence and relapse-like drinking in adulthood. AO’s effects were associated with its modulation of the HPA axis (elevated serum corticosterone), restoration of amygdala oxidative balance, BDNF levels, and attenuation of neurodegeneration. These findings suggest that AO’s neuroprotective properties could offer a potential therapeutic avenue for reducing ethanol/stress-induced brain damage and addiction. Further research is needed to explore its mechanisms and therapeutic potential in alcohol use disorders.

## 1. Introduction

Adolescence marked by binge-like alcohol exposure can lead to long-term adverse consequences, including an elevated risk of developing alcohol use disorder (AUD) [1,2,3]. Research indicates that early initiation and high levels of alcohol consumption during this critical developmental period can disrupt normal brain maturation, affecting cognition and reward sensitivity [4,5,6]. The adolescent brain is particularly susceptible to alcohol’s harmful effects, which may lead to persistent behavioral issues and cognitive impairments into adulthood, as evidenced by studies on both human populations and rodent models [6].

Stress is a significant predictor of increased alcohol use and related disorders, with adolescent stressors exacerbating these risks. Stress exposure during adolescence can amplify alcohol consumption and exacerbate emotional and behavioral disturbances, although results are mixed on the exact nature of stress’s impact on alcohol intake in adults [7,8]. Stress and alcohol exposure during adolescence interact in complex ways, affecting impulsivity, behavioral inhibition, and stress sensitivity, with these effects potentially persisting into adulthood [9].

Emotional dysregulation during alcohol withdrawal and/or stress exposure is largely attributed to changes within the extended amygdala [10,11,12]. In general, the central (CeA), medial, and basolateral nuclei of the amygdala have been shown to be heavily implicated in the processing of anxiety and addiction [13,14], and binge-like alcohol-induced changes within CeA are implicated in the emotional disturbances that can occur during alcohol abstinence [15]. Chronic or binge-like alcohol exposure leads to alterations in amygdala morphology and neurochemistry, including increased oxidative stress and disrupted signaling pathways, particularly involving the brain-derived neurotrophic factor (BDNF) [16,17,18].

Previously, we found that adolescent ethanol binge-like exposure [19] or chronic stress [20] exposure, increases oxidative stress and neurodegeneration in the rat’s amygdala. Stress-induced changes in the amygdala during alcohol withdrawal are linked to emotional dysregulation and negative affect, but the combined effects of adolescent binge alcohol exposure and chronic stress on the amygdala function remain underexplored [10]. These challenges may significantly limit the efficacy of conventional pharmacological treatments for AUD, particularly in cases where stress-induced psychiatric comorbidities are present, highlighting the need for alternative therapeutic approaches.

Argan oil (AO) is obtained from the argan fruit of *Argania spinosa* L., an endemic tree located mainly in south-western Morocco [21]. Chemical analysis of AO shows that it is mainly rich in polyunsaturated fatty acids (80%), especially oleic (44.8%) and linoleic acids (33.7%), and antioxidants such as coenzyme Q10 (CoQ10), melatonin, tocopherols, and polyphenols [22,23]. In this respect, AO is emerging as a promising therapeutic agent. Currently, it is becoming largely acclaimed for a variety of health and wellness virtues [24]. Results of various studies that were completed in vitro or on human and animal models have stated that AO possesses choleretic, hepatoprotective, cardiovascular-protective, chemopreventive, antioxidant, anti-inflammatory, anti-malarial, anti-diabetic, anti-atherosclerotic, anti-hypercholesterolemic, anti-hypertensive, anti-proliferative, and anti-carcinogenic properties [25,26,27].

Previous studies conducted in our laboratory (Biology and Health Laboratory), upheld—for the first time—that AO may exhibit neuroprotection against certain specific aspects of adolescent intermittent ethanol intoxication (IEI) and unpredictable mild stress (UMS)-induced addictive-like and depressive-like behaviors, respectively (review in [28]). First, in the adolescent IEI model we found that AO pre-treatment at a 10 mL/kg/day dose improves the cognitive abilities in rats and attenuates oxidative stress in both the prefrontal cortex (PFC) and the hippocampus [29]. Second, in adult rats previously exposed to IEI during early adolescence, we demonstrated that AO supplementation (10 mL/kg/day) significantly decreases voluntary ethanol consumption. Accordingly, withdrawal syndrome and anxiety-like behaviors were remarkably reduced by AO pre-treatment after ethanol removal.

Moreover, because of the low ethanol consumption, IEI-elicited oxidative stress in the PFC, hippocampus, and amygdala brain regions, and neurodegeneration and neuronal loss in the CA3 hippocampal subregion, have been counteracted by AO pre-treatment [19]. During these two studies, the supplementation with AO was before, during, and after IEI exposure. Thus, certain beneficial effects of AO have been attributed—at least in part—to the neuroprotective/preventive properties of this vegetable oil. The current study aims to evaluate the efficacy of AO in mitigating the adverse effects of early adolescent binge alcohol exposure combined with chronic stress. Specifically, it investigates whether AO treatment can reverse the negative behavioral outcomes, reduce oxidative stress in the amygdala, normalize corticosterone (CORT) levels, and attenuate neuronal damage and BDNF alterations in the CeA induced by these stressors and alcohol exposure.

## 2. Results

### 2.1. Body Weight Gain

A two-way RM-ANOVA on body weight gain, considering the group and time as factors in water- or AO-treated rats, revealed significant effects of group (water: F (2.3) = 170.3, *p* < 0.001; AO: F (1.4) = 87.36, *p* < 0.001), time (water: F (17.7) = 151.8, *p* < 0.001; AO: F (17.7) = 235.5, *p* < 0.001), and a group–time interaction (water: F (51.2) = 1.97, *p* = 0.0004; AO: F (51.2) = 2.76, *p* < 0.001). The time effect was attributed to a progressive body weight increase throughout the experiment. At the end of the treatment period (PND127), body weight gain was 244 ± 7.8 g in the Ctrl group, 239 ± 9.1 g in the IEI group, 239 ± 9.0 g in the UMS group, and 197 ± 8.1 g in the IEI + UMS group for water-treated animals. In AO-treated animals, weight gains were 335 ± 22.2 g for Ctrl, 314 ± 15.1 g for IEI, 305 ± 3.8 g for UMS, and 220 ± 2.1 g for IEI + UMS. Post-hoc analysis indicated that the group–time interaction was mainly due to a significant weight loss in the IEI/UMS group compared to the control in AO-treated animals (*p* < 0.001). Additionally, each condition in the AO cohort showed significantly higher body weight compared to the corresponding water-treated condition (*p* < 0.001 for all comparisons).

### 2.2. Home-Cage Voluntary Ethanol Consumption in a 2-Bottle Free Choice Test

**Figure 1:**
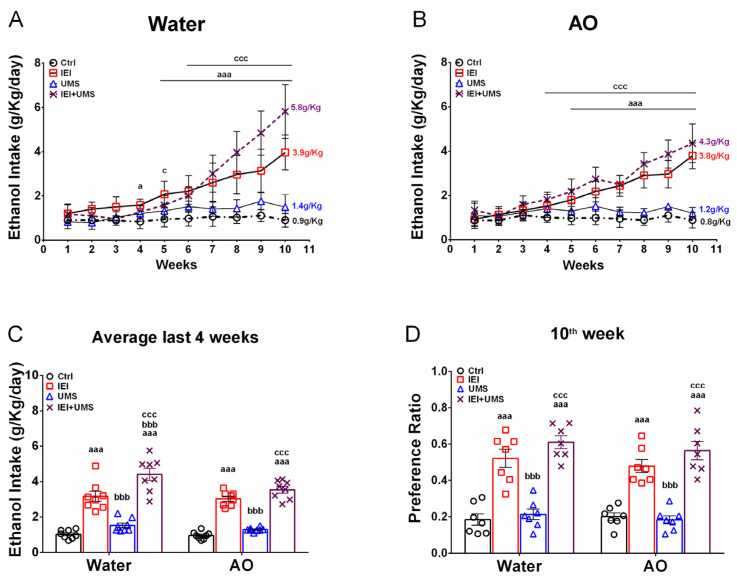
Home-cage voluntary ethanol consumption in a 2-bottle free choice test. (**A**,**B**) Weekly intermittent 20% ethanol intake in water and AO-treated cohorts, respectively. (**C**) Average ethanol intake over the last 4 weeks of the experiment. (**D**) Average preference ratio over the last week of the experiment of Ctrl (*n* = 12), IEI (*n* = 12), UMS (*n* = 12) and IEI/UMS (*n* = 12) with/without AO treatment. Results were expressed as mean ± SEM. (**A**,**B**): ^aaa^ *p* < 0.001, IEI vs. Ctrl; ^ccc^ *p* < 0.001, Ctrl vs. IEI/UMS. (**C**,**D**): ^aaa^ *p* < 0.001 compared with Ctrl; ^bbb^ *p* < 0.001 compared with IEI; ^ccc^ *p* < 0.001 compared with UMS, according to RM 3-way or 2-way ANOVA, followed by a Tukey’s test.

In the 2-bottle free choice test, the effects of adolescent IEI and UMS on voluntary ethanol consumption under intermittent 20% ethanol access were assessed (Figure 1A,B). Separate 3-way RM-ANOVAs (time × IEI × UMS) were conducted for water- and AO-treated rats, revealing significant effects of time (water: F (9.9) = 59.89, *p* < 0.001; AO: F (9.9) = 50.52, *p* < 0.001), IEI (Water: F (1.9) = 473.8, *p* < 0.001; AO: F (1.9) = 637.4, *p* < 0.001), and UMS (Water: F (1.9) = 27.99, *p* < 0.001; AO: F (1.9) = 57.11, *p* < 0.001), but no interaction between factors in both cohorts. Post-hoc analyses revealed a significant increase (*p* < 0.05) in ethanol intake in the IEI and IEI/UMS groups compared to controls. In water-treated rats (Figure 1A), this increase was observed from week 4 or 5, with final ethanol intake reaching 3.96 ± 0.28 g/kg/24 h for IEI and 5.81 ± 0.42 g/kg/24 h for IEI/UMS rats, while controls maintained a low intake (0.90 ± 0.11 g/kg/24 h). In AO-treated rats (Figure 1B), ethanol intake increased from week 5, reaching 3.78 ± 0.20 g/kg/24 h for IEI and 4.35 ± 0.30 g/kg/24 h for IEI/UMS rats, while controls remained at 0.88 ± 0.12 g/kg/24 h.

Additionally, no significant difference in ethanol intake was observed between Ctrl and UMS groups in either cohort, with UMS rats consuming 1.20 ± 0.08 g/kg/24 h (Water cohort) and 1.48 ± 0.19 g/kg/24 h (AO cohort). Ethanol consumption was significantly lower in UMS compared to IEI/UMS rats (*p* < 0.001) during the last 3 weeks of the experiment for both water and AO cohorts. During the last three weeks, the IEI/UMS water-treated rats displayed a significantly higher ethanol intake than IEI rats (*p* < 0.001), but no significant difference between these groups was found under AO treatment.

To further explore AO treatment effects, average ethanol intake over the last 4 weeks was compared across groups (Figure 1C). A three-way ANOVA (AO × IEI × UMS) confirmed significant effects of IEI (F (1.1) = 311.5, *p* < 0.001) and UMS (F (1.1) = 23.91, *p* < 0.001), but no effect of AO treatment (F (1.1) = 6.197, *p* = 0.0603) and no interaction between factors. Ethanol preference (Figure 1D) also showed a significant effect of IEI (F (1.1) = 188, *p* < 0.001), but no effect of UMS (F (1.1) = 3.344, *p* = 0.0737), AO treatment (F (1.1) = 1.009, *p* = 0.3202), or interaction between factors.

### 2.3. EWS Score after 2–72 H of Abstinence

**Figure 2:**
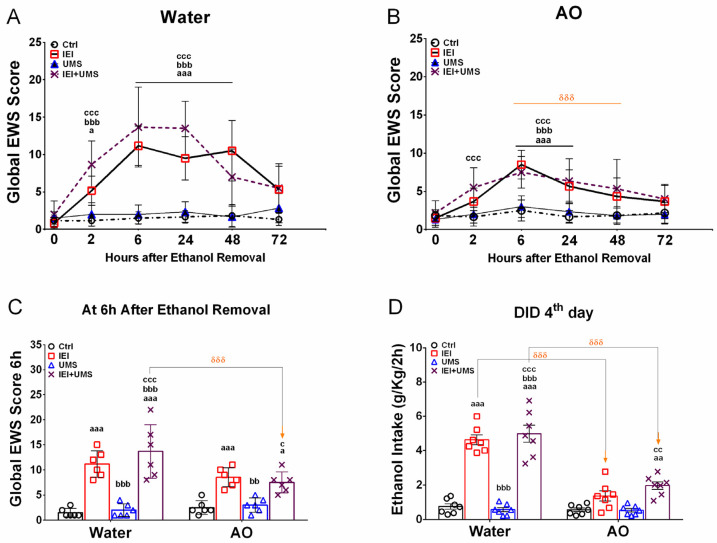
Ethanol withdrawal signs and binge-like ethanol drinking in the dark after 72 h of abstinence. (**A**,**B**) Global EWS (sum of somatic withdrawal scores across the five behavioral signs) measured between 2 and 72 h after the removal of the 20% *v*/*v* ethanol solution, in water and AO-treated rats, respectively. (**C**) Average of global EWS scores measured at the 6th and the 24th hours of ethanol removal. (**D**) 20% *v*/*v* ethanol consumption (g/kg/2 h) on the 4th day of the drinking in the dark test in water- and AO-treated Ctrl (*n* = 10), UMS (*n* = 11), IEI (*n* = 11), and IEI/UMS (*n* = 11) groups. The values represent the mean ± SEM. (**A**,**B**): ^a^ *p* < 0.05, ^aaa^ *p* < 0.001, IEI vs. Ctrl; ^bbb^ *p* < 0.001, Ctrl vs. IEI/UMS; ^ccc^ *p* < 0.001, UMS vs. IEI/UMS. (**C**,**D**): ^a^ *p* < 0.05, ^aa^ *p* < 0.01, ^aaa^ *p* < 0.001 compared with Ctrl; ^bb^ *p* < 0.01, ^bbb^ *p* < 0.001 compared with IEI; ^c^ *p* < 0.05, ^cc^ *p* < 0.01, ^ccc^ *p* < 0.001 compared with UMS. ^δδδ^ *p* < 0.001 AO effect, according to RM 3-way or 2-way ANOVA, followed by Tukey’s test. AO: Argan oil, EWS: Ethanol withdrawal signs.

A separate 3-way RM-ANOVA of the global EWS score using IEI, UMS, and hours as factors in water-treated (Figure 2A) and AO-treated (Figure 2B) rats, showed significant main effects of IEI (water: F (1.5) = 250.1, *p* < 0.001; AO: F (1.5) = 74.64, *p* < 0.001) and hours (water: F (5.5) = 20.85, *p* < 0.001; AO: F (5.5) = 10.47, *p* < 0.001), with no significant interaction between factors (water: F (1.5) = 1.982, *p* = 0.0861; AO: F (5.5) = 0.583, *p* = 0.7129). The UMS condition had a significant effect only in water-treated rats (water: F (1.5) = 33.14, *p* < 0.001; AO: F (1.5) = 1.818, *p* = 0.1801). Post-hoc analysis indicated that ethanol withdrawal led to an increase in global EWS scores in both water-treated IEI and IEI/UMS rats, with notable differences between the two groups during abstinence. Water-treated IEI rats showed a significant increase in EWS scores at 2, 6, and 24 h of abstinence (*p* = 0.015, *p* < 0.001, *p* < 0.001, respectively) compared to controls. In contrast, water-treated IEI/UMS rats exhibited persistently elevated EWS scores throughout the abstinence period (*p* < 0.001 at all-time points). Additionally, IEI/UMS rats had significantly higher EWS scores compared to IEI rats at 24 and 48 h of withdrawal (*p* = 0.0473 and *p* < 0.001, respectively).

In the IEI and IEI/UMS rats, the combined sum of withdrawal signs (posture, gait, agitation, tail stiffness, and stereotypical behavior) progressively increased from 5.1 ± 0.7 and 8.6 ± 1.2 at 2 h to 4.8 ± 1.0 and 12.83 ± 1.2 at 24 h, peaking at 6 and 24 h with scores of 11.1 ± 1.0 and 13.8 ± 1.4, respectively, confirming a significant overall severity of withdrawal. All individual withdrawal ratings at 6 hours’ post-ethanol removal were significantly elevated in IEI and IEI/UMS rats compared to water-treated controls (*p* < 0.05).

AO treatment during voluntary ethanol consumption significantly reduced withdrawal signs in both IEI and IEI/UMS animals compared to water-treated controls (Figure 2B). Post-hoc Tukey tests revealed that the EWS score increases in AO-treated IEI and IEI/UMS rats compared to controls that were only significant at the 6th and 24th hours (*p* < 0.001). Furthermore, no significant differences in EWS scores were found between AO-treated IEI and IEI/UMS rats at different withdrawal time points. A three-way ANOVA on EWS scores at 6 and 24 h (Figure 2C) showed the main effects of AO treatment (F (1.1) = 36.62, *p* < 0.001), IEI (F (1.1) = 294.3, *p* < 0.001), UMS (F (1.1) = 7.566, *p* = 0.0089), and an AO × IEI × UMS interaction (F (1.1) = 4.205, *p* = 0.0469). Post-hoc analysis confirmed that AO-treated IEI and IEI/UMS rats had significantly lower EWS scores than their water-treated counterparts (*p* < 0.001), with maximum EWS scores of 6.6 ± 1.1 and 7.3 ± 1.1, respectively, during abstinence. Individual withdrawal ratings measured at 6 hours’ post-ethanol removal were significantly lower in AO-treated rats than in water-treated rats. These results suggest that AO treatment during ethanol consumption attenuates or prevents the manifestation of ethanol withdrawal signs in alcohol/stress-exposed rats.

### 2.4. Binge-like Drinking in the DID Test

Following ethanol withdrawal, rats were subjected to a restricted free-choice 20% ethanol access (vs. tap water) in the dark phase for 4 days. As shown in Figure 2D, a three-way ANOVA on average ethanol consumption over 2 h revealed significant effects of IEI (F (1.1) = 211.4, *p* < 0.001) and AO treatment (F (1.1) = 80.89, *p* < 0.001), but no effect of UMS (F (1.1) = 1.043, *p* = 0.3123) and no interaction between factors (F (1.1) = 0.0185, *p* = 0.8922). Post-hoc analysis showed a significant increase in binge-like ethanol intake in water-treated IEI and IEI/UMS groups compared to both the water-treated control and UMS groups (*p* < 0.001).

In contrast, AO treatment eliminated these differences, with no significant difference in binge-like ethanol intake between IEI, UMS, and control groups under AO conditions. Moreover, AO-treated IEI and IEI/UMS rats displayed a significant reduction in ethanol intake compared to water-treated IEI and IEI/UMS rats (*p* < 0.001 for both). While water-treated IEI and IEI/UMS rats exhibited a marked increase in ethanol intake (4.6 ± 0.2 g/kg/2 h and 4.9 ± 0.5 g/kg/2 h, respectively), AO-treated rats showed much lower consumption (1.3 ± 0.2 g/kg/2 h and 1.9 ± 0.2 g/kg/2 h, respectively), approaching levels seen in healthy control rats (0.7 ± 0.1 g/kg/2 h). These results suggest that AO supplementation, by reducing ethanol withdrawal signs during abstinence, counteracts and/or prevents relapse-like drinking in alcohol- and stress-exposed rats.

### 2.5. Neurodegeneration Histological Analysis in CeA

**Figure 3:**
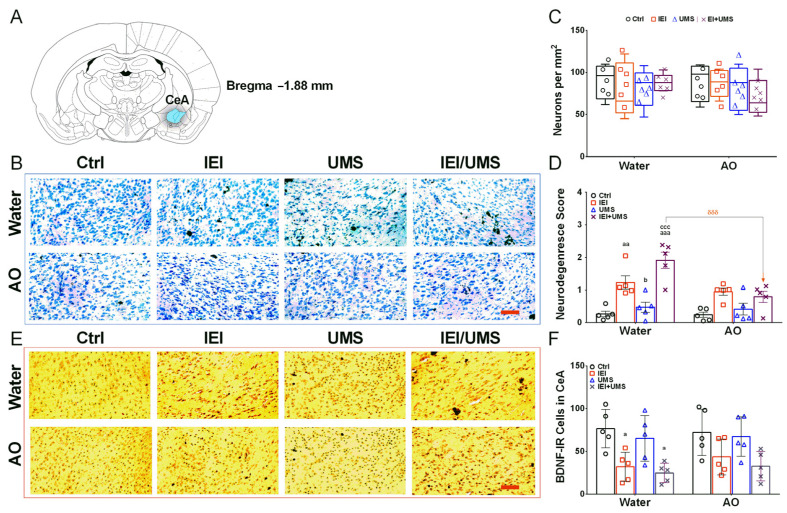
Histologic neurodegeneration and BDNF immunoreactivity analysis in the CeA. (**A**) Brain anatomical positions of the CeA, relative to Bregma −1.88 mm from the rat brain atlas [30]. (**B**) Representative images of Nissl-stained forebrain CeA sections. (**C**) Number of neurons per mm^2^ assessed by cell counting and (**D**) Semi-quantitative analysis of neurodegeneration (score) in rats CeA sections. (**E**) Representative images showing BDNF—immunolabelling observed in the CeA sections. (**F**) Positive BDNF-IR cell counts in the CeA sections in Ctrl (*n* = 5), IEI (*n* = 4), UMS (*n* = 5) and IEI/UMS (*n* = 5) groups under water or AO treatment. Results are expressed as mean ± SEM from 5 animals per group. ^a^ *p* < 0.05, ^aa^ *p* < 0.001 and ^aaa^ *p* < 0.001 compared to Ctrl group; ^b^ *p* < 0.05 compared to the IEI group; ^ccc^ *p* < 0.001 compared to the UMS group; ^δδδ^ *p* < 0.001 AO effect (water-treated IEI/UMS vs. AO-treated IEI/UMS). The data of neuron counting are illustrated as box-and-whisker plots. Scale bar = 50 µm. CeA: the central nucleus of the amygdala, AO: Argan oil, BDNF-IR cells: brain-derived neurotrophic factor—immunoreactive cells.

As shown in Figure 3, semi-quantitative analysis of neurodegeneration in the CeA was conducted using two methods: visual scoring (Figure 3D) and neuronal counting per mm^2^ (Figure 3C). Kruskal–Wallis ANOVA followed by Dunn’s post-hoc test revealed no significant difference in the number of neurons between groups in either the water or AO treatment cohorts. However, significant neurodegeneration was observed in water-treated IEI rats, and was highly significant in water-treated IEI/UMS rats compared to water-treated control rats (scores: *p* = 0.0053 and *p* < 0.001, respectively). Additionally, both IEI and IEI/UMS rats exhibited significant neuronal loss compared to UMS rats in the water group (*p* = 0.0282 and *p* < 0.001, respectively). No significant differences in neurodegeneration scores were found between groups in the AO-treated cohort. However, chronic AO treatment during ethanol consumption significantly reduced neuronal loss in the AO-treated IEI/UMS rats compared to the water-treated IEI/UMS rats (score: *p* < 0.001).

### 2.6. Immunohistochemistry Analysis of BDNF-IR Cells in CeA

Significant changes in BDNF-IR cell levels of the CeA were detected between groups (Figure 3F). While a three-way ANOVA did not show significant interaction between factors (IEI × UMS × AO: F (3.3) = 0.265, *p* = 0.8496) or an AO treatment effect (F (1.3) = 0.391, *p* = 0.5358), it revealed a main effect of IEI/UMS (F (3.3) = 10.74, *p* < 0.001) on CeA BDNF-IR cell levels. BDNF-IR staining was localized in neuronal cytoplasm and sometimes extended into apical fibers. Post-hoc Tukey tests indicated a significant increase in this parameter in the CeA of IEI and IEI/UMS rats compared to the water-treated controls (*p* = 0.0411 and *p* = 0.0108, respectively). However, no significant differences in this parameter were found after chronic AO treatment compared to AO-treated controls or UMS rats.

In summary, these findings suggest that AO supplementation during voluntary alcohol consumption mitigates the long-lasting brain damage caused by adolescent IEI and UMS exposure by reducing neurodegeneration and normalizing BDNF levels in the amygdala.

### 2.7. CORT Levels in Plasma

Plasma CORT levels were measured from tail blood samples collected between 6:00 pm and 7:00 pm after the final drinking session. As shown in Table 1, a three-way ANOVA revealed significant effects of IEI (F (1.1) = 22.09, *p* < 0.001) and UMS (F (1.1) = 4.701, *p* = 0.0377), but no significant effect of AO treatment (F (1.1) = 3.746, *p* = 0.0618) and no interaction between factors (F (1.1) = 0.0353, *p* = 0.8521). Post-hoc analysis indicated that, following 11 weeks of intermittent 20% ethanol access, plasma CORT levels were significantly higher in IEI rats compared to control rats in the water cohort (*p* = 0.0329). Additionally, the combination of IEI and UMS conditions resulted in even higher CORT levels compared to the control group (*p* = 0.0098). In contrast, no significant differences in plasma CORT levels were observed between groups in the AO-treated cohort.

### 2.8. Oxidative Stress in Amygdala

Following 11 weeks of ethanol drinking (on PND 121), separate animals from each experimental group, in both water and AO treatment cohorts, were used to measure MDA levels, NO levels, CAT, and SOD activities in the amygdala. The results are summarized in Table 1.

#### 2.8.1. MDA Levels

ANOVA analysis of MDA levels in the amygdala revealed significant effects of both AO treatment (F (1.1) = 8.864; *p* = 0.005) and IEI condition (F (1.1) = 31.6; *p* < 0.001), with no significant effect of the stress condition (F (1.1) = 0.3506; *p* = 0.5579) or interaction between factors (F (1.1) = 0.3847; *p* = 0.5395). Post-hoc analysis showed that adolescent binge-like ethanol exposure significantly increased MDA levels in the amygdala of IEI and IEI/UMS rats compared to Ctrl rats (*p* = 0.0129 and *p* = 0.0025, respectively). IEI/UMS rats also had higher MDA levels compared to UMS rats (*p* = 0.0226). AO treatment reduced MDA levels in IEI/UMS rats and nearly reduced them in IEI rats, bringing MDA values closer to those of the Ctrl group (*p* = 0.0493 and *p* = 0.0685, respectively). No significant differences were observed between groups in the AO cohort.

#### 2.8.2. NO Levels

On the NO levels, ANOVA indicated a significant effect of the IEI condition (F (1.1) = 27.19; *p* < 0.001), but no effect of AO treatment (F (1.1) = 2.026; *p* = 0.6143), UMS condition (F (1.1) = 0.9398; *p* = 0.3396), or interaction between factors (F (1.1) = 0.4117; *p* = 0.5257). Inter-group analysis showed a significant increase in NO levels in IEI and IEI/UMS rats compared to Ctrl rats (*p* = 0.0347 and *p* = 0.0098, respectively). AO treatment did not significantly reduce NO levels in these groups, and no significant differences were observed between groups in the AO cohort.

#### 2.8.3. SOD Activity

Regarding the SOD activity, ANOVA demonstrated significant effects of the IEI condition (F (1.1) = 13.49; *p* = 0.0009) and AO treatment (F (1.1) = 8.789; *p* = 0.0057), with no effect of the UMS condition (F (1.1) = 1.306; *p* = 0.2615) or interaction between factors (F (1.1) = 0.0520; *p* = 0.8211). Post-hoc analysis revealed decreased SOD activity in IEI and IEI/UMS rats compared to Ctrl rats (*p* = 0.0461 and *p* = 0.0048, respectively). AO treatment significantly increased SOD activity in IEI/UMS rats compared to water-treated IEI/UMS rats (*p* = 0.0379). However, no significant differences were found between different groups in the AO cohort.

#### 2.8.4. CAT Activity

ANOVA of the CAT activity showed significant effects of the IEI condition (F (1.1) = 15.11; *p* = 0.0005) and AO treatment (F (1.1) = 5.743; *p* = 0.0226) on CAT activity, with no effect of the UMS condition (F (1.1) = 1.084; *p* = 0.3057) or interaction between factors (F (1.1) = 0.3589; *p* = 0.5533). Tukey post-hoc analysis revealed that binge-like ethanol exposure combined with stress significantly decreased CAT activity in the amygdala of IEI/UMS rats compared to Ctrl rats (*p* = 0.0260). AO treatment slightly improved CAT activity in both IEI and IEI/UMS rats, approaching the levels seen in the healthy Ctrl group.

In sum, the results indicate significant alterations in oxidative status in the amygdala of IEI and IEI/UMS rats. Chronic AO treatment counteracts these changes, with no significant effects observed in the UMS group, where alcohol intake was low throughout the experiment.

## 3. Discussion

The study reveals that combined adolescent forced binge-like ethanol exposure and mild stress lead to increased voluntary ethanol consumption, withdrawal signs, and relapse-like drinking in adulthood, along with elevated plasma CORT levels and neuronal damage in the amygdala, including neurodegeneration, oxidative stress, and reduced BDNF levels. These effects were specific to the IEI and IEI/UMS conditions, with no impact from UMS alone. In contrast, AO treatment at 15 mL/kg/day, while minimally affecting ethanol consumption, effectively mitigates these adverse outcomes by reducing withdrawal signs, relapse-like drinking, normalizing CORT and BDNF levels, and alleviating oxidative stress in the amygdala following adolescent alcohol-stress exposure.

1.1. Effectiveness of the forced binge-like ethanol and mild stress alternate exposure during adolescence

Studies have demonstrated that adolescent binge-like ethanol exposure and/or stress have long-lasting harmful effects on adolescent brain maturation and development [4]. The IEI model used in this study simulates binge-like ethanol exposure during half of the adolescent period (15 days out of the 30-day period), with 2-day withdrawal intervals [31]. This model effectively induces increased ethanol consumption in various rodent strains [16,25,32], which is consistent with our findings in Wistar rats. However, the mechanisms through which adolescent binge ethanol exposure leads to increased ethanol intake in adulthood remain unclear. This work aims—first—to determine if combining adolescent IEI exposure with unpredictable mild stress (UMS) further exacerbates ethanol consumption and withdrawal signs. Results show that UMS significantly amplifies ethanol consumption in IEI-exposed rats, with intake rising from 3.93 ± 0.28 g/kg/24 h in non-stressed IEI rats to 5.81 ± 0.42 g/kg/24 h in stressed IEI rats. Additionally, IEI/UMS rats display persistent and significant withdrawal signs throughout the abstinence period, along with increased relapse-like drinking after ethanol deprivation. While IEI rats show a significant increase in the global EWS score compared to Ctrl animals only during 6th, 24th, and 48th h withdrawal time points, the IEI/UMS rats displayed a significant increase in this parameter throughout the abstinence period (72 h). These findings suggest that UMS exacerbates ethanol consumption escalation and withdrawal signs, facilitating a transition to dependence-like drinking in IEI-exposed rats.

Results from IEI-treated rats align with our recent findings on ethanol consumption, withdrawal signs, and locomotor activity [19]. Adolescent IEI-increased voluntary ethanol consumption in a 2-bottle choice test with 10% ethanol, resulting in higher EWS scores and anxiety-like behaviors after 72 h of withdrawal, but did not affect locomotor activity. This is consistent with previous studies indicating that early binge-like ethanol exposure enhances ethanol consumption and withdrawal signs in adulthood [33,34,35]. The effects were more pronounced when IEI was combined with UMS during adolescence. Negative affect strongly influences the withdrawal/abstinence phase of addiction, with individuals with AUD often reporting stress, depression, and anxiety as relapse factors [10]. In the DID test, IEI and IEI/UMS conditions significantly increased binge-like ethanol intake after 4 days of abstinence, with IEI and IEI/UMS rats consuming 4.6 ± 0.2 g/kg and 4.9 ± 0.5 g/kg of 20% ethanol per 2 h, respectively. This suggests a robust reinstatement of drug-seeking behavior and relapse-like drinking. The UMS appears to enhance ethanol intake in the IEI model, possibly by increasing ethanol’s reinforcing properties and withdrawal effects.

The underlying mechanisms of this interaction remain unclear but may involve stress’s impact on ethanol’s reinforcing properties or its enhancement of withdrawal-related dysphoria. Studies indicate that anxiety and depression during ethanol withdrawal contribute to increased drug-seeking behavior and stress responses [36,37,38,39,40]. Our findings suggest that these consumption patterns can produce enduring affective disturbances during abstinence and relapse-like drinking, exacerbated by UMS exposure. Mechanistically, the transition from regulated to excessive drinking involves brain stress systems and amygdala dysregulation [39]. Neuroadaptations in CRF, dynorphin, and glucocorticoid signaling have been linked to escalated drinking [41,42]. Stress exposure may exacerbate these adaptations, leading to increased ethanol consumption and relapse. We observed that IEI exposure followed by intermittent 20% ethanol drinking resulted in HPA axis hyperactivity, with increased plasma CORT levels in IEI and even higher levels in IEI/UMS rats. Previous studies highlight correlations between baseline CORT, anxiety-like behavior, and ethanol intake/preference [43]. Additionally, central CRF receptor antagonism reduces ethanol self-administration and anxiety-like behavior during withdrawal [44].

CORT-related plasticity has been observed in various brain regions, including the CeA, which plays a crucial role in anxiety, stress, and reward regulation. Increased CeA activation correlates with heightened anxiety-like behaviors and is associated with stress and affective disorders in humans [32]. Stress exposure in vivo leads to CeA plasticity, characterized by increased anxiety, ethanol drinking, dendritic hypertrophy, spine density, and dysregulation of neurotrophic factors [45,46,47]. Binge-like ethanol exposure and mild stress may induce similar changes in the CeA, mediated by HPA axis dysregulation. Consistent with our previous study [19], we observed that adolescent binge-like ethanol intoxication, followed by 10 weeks of intermittent 20% ethanol drinking, significantly increased CeA neurodegeneration scores 1 day post-ethanol removal, with more pronounced effects under UMS conditions. However, UMS alone did not alter CeA neurodegeneration or neuronal numbers compared to control rats. Molecular alterations and neurodegeneration in key affective and reward-related brain regions such as the nucleus accumbens (NAc), CeA, and prelimbic cortex have been well documented [48,49]. Our data suggest that UMS exacerbates neurodegeneration in IEI-exposed rats. Additionally, decreased BDNF levels were found in the CeA of both IEI and IEI/UMS rats, which may contribute to negative affective behaviors.

BDNF, a neurotrophic factor involved in neurogenesis, synaptic plasticity, and HPA axis regulation [50,51], is critical for stress and reward regulation and plays a role in AUD pathophysiology. Our results indicate that reduced BDNF signaling in the CeA may mediate emotional disturbances and relapse-like drinking in IEI and IEI/UMS rats. Moreover, ethanol withdrawal induced oxidative stress in the amygdala of IEI rats, evidenced by increased MDA and NO levels and decreased CAT and SOD activities. This oxidative imbalance was more pronounced in IEI/UMS rats. Ethanol exposure is known to induce oxidative stress and subsequent brain damage, which is linked to neuroinflammation, neurotoxicity, and neurodegeneration [52]. Our findings suggest that mild stress during adolescence enhances voluntary ethanol drinking and withdrawal symptoms through increased plasma CORT levels, amygdala neurodegeneration, and oxidative stress, while downregulating BDNF levels in the CeA brain subregion.

The following paragraph will discuss potential therapeutic effects of AO supplementation against the ethanol-stress interaction that induces addictive-like disorders.

1.2. Effectiveness of AO treatment against binge-like ethanol/mild stress-induced behaviors, biochemical and histochemical disabilities

Our study investigates whether AO treatment during ethanol consumption affects long-lasting behavioral changes, neurodegeneration, neurotrophic, and oxidative damage in the amygdala induced by adolescent IEI/UMS. Rats were administered AO (15 mL/kg/day) for 11 weeks starting 1 day after ethanol/stress exposure. Our results show that AO supplementation resulted in a low decrease in voluntary ethanol consumption and ethanol preference, especially in IEI/UMS received rats. Indeed, while water-treated IEI/UMS animals showed a high and strong significant increase escalation in their ethanol consumption, AO-treated IEI/UMS rats displayed a low but significant increase in their ethanol intake under 10 weeks of intermittent 20% ethanol access. During the last 4 weeks of a two-bottle free choice test, water-treated IEI/UMS animals reached a mean ethanol intake of 4.40 ± 0.33 g/kg/24 h (around of 53% ethanol preference) whereas AO-treated IEI/UMS animals displayed a mean ethanol intake of 3.53 ± 0.18 g/kg/24 h (around of 46% ethanol preference) (Figure 1C). Nevertheless, no effect of AO dietary was detected in this parameter when water-treated IEI rats are compared with AO-treated IEI ones. Those rats reached a mean ethanol intake of 3.16 ± 0.28 g/kg/24 h and 3.02 ± 0.14 g/kg/24 h, respectively, during the last 4 weeks of the experiment.

In contrast, we previously found that AO pre-treatment at 10 mL/kg/day dose decreased significantly the voluntary ethanol consumption induced by adolescent IEI in rats [19]. This beneficial effect may be due to the longer duration of AO supplementation (14 weeks vs. 11 weeks in the current work), which took place before, during and after ethanol intoxication. Herein, despite the increase in the administered AO dose (from 10 mL/kg/day to 15 mL/kg/day), the treatment with this vegetable oil after the IEI exposure, showed a slight influence on voluntary ethanol consumption. Thus, the benefits of AO may be primarily preventative rather than therapeutic and confirm the effectiveness of the early supplementation procedure (starting at weaning (PND21)) used in our previous studies [19,20,29]. Also, these results suggest a possible effect of AO orally administered on the pharmacokinetics of ethanol. Unfortunately, without measuring the blood ethanol concentration in the AO-treated IEI rats, this last suggestion remains hypothetical and constitutes a limitation of our study. Another possible explanation is the difference in the alcohol self-administration procedures used in these studies. As shown by others using rats [53] and mice [54], ethanol consumption was significantly elevated when the drug was made available on an intermittent rather than continuous schedule. It has been suggested that forced abstinence during the intermittent ethanol access procedure enhanced the motivation for voluntary ethanol consumption.

Following 10 weeks of voluntary ethanol drinking, we tested the possible “therapeutic” effect of AO against the ethanol withdrawal-induced physical dependence in rats, with a history of IEI and/or UMS. Accordingly, we previously showed that AO produced a prominent inhibitory effect on withdrawal signs in both of IEI and IEI/UMS received rats [19,20]. Indeed, during 72 h of abstinence AO treatment reduced significantly the EWS score in the IEI/UMS as compared to the water-treated IEI/UMS or to the AO-treated IEI ones. Thus, by reducing ethanol intake in rats under IEI and UMS conditions, the AO treatment attunes the manifestation of EWS after ethanol removal. Notably, we found that AO treatment during voluntary ethanol consumption significantly decreases the relapse-like drinking during ethanol withdrawal in rats with a history of adolescent alcohol-stress exposure. In the DID test conducted 4 days after ethanol removal, AO-treated IEI and IEI/UMS showed a highly significant decrease in the binge-like 20% ethanol intake, as compared with the water-treated IEI and IEI/UMS rats, respectively (*p* < 0.001 for both comparisons). In addition, no significant difference in this parameter was detected between the IEI, the UMS, and the Ctrl groups when those rats are previously treated with AO. Overall, these findings suggest that AO treatment, by reducing ethanol withdrawal signs during abstinence, counteracts and/or prevents a subsequent relapse-like drinking in the adolescent alcohol/stress-exposed rats.

In the present study, the reason that AO reduced voluntary ethanol intake, withdrawal signs, and subsequent relapse-like drinking—especially in IEI/UMS received rats—was unclear. However, several plausible explanations can be proposed, such as modulation of the HPA axis, brain morphological/functional plasticity and pro-oxidant/antioxidant balance. Mostly, phytochemicals demonstrate anti-addictive activities by different mechanisms such as alteration of brain monoamine levels, neurotrophic and neuroprotective effects via BDNF-CREB signaling pathway, modulation of the HPA axis and antioxidant system [28,55]. Herein, alcohol-stress induced hyperactivity of the HPA was studied by determining the elevated serum CORT concentrations at the end of 11 weeks of AO treatment. Clinical and pre-clinical studies evidenced that several addicted patients were observed with hypercortisolism during the withdrawal period and suggested that anti-depressant drugs suppressed the serum CORT levels and reduced ethanol intake by normalization of the HPA axis [56]. Importantly, we show that AO supplementation improved the plasma CORT levels in the IEI and IEI/UMS received rats. Indeed, no significant difference in this parameter was found between the groups in the AO-treated cohort. Previously, we found that prolonged AO supplementation (10 mL/kg/day) reduced plasma CORT levels in rats exposed to UMS and returned its values to those of the healthy control group [51]. We suggest that supplementation with AO during voluntary ethanol consumption acted as an “anti-depressant drug” in the normalization of the HPA axis, and by reducing plasma CORT levels AO treatment counteracts affective disorders that emerged during the ethanol withdrawal period.

On the other hand, our results show that adolescent ethanol-stress exposure down- regulated the BDNF levels in the CeA. However, immunohistochemistry analysis has confirmed the upregulation of this parameter by AO supplementation. Thus, no significant changes in the density of BDNF-IR neurons in the CeA were found between AO-treated rats under Ctrl, IEI, UMS, and IEI/UMS conditions. Numerous compounds present in AO, including phytosterols, schottenol, spinasterol, polyphenols, tocopherols, and polyunsaturated fatty acids (PUFAs) have been widely characterized as neuroprotective agents. These compounds, which are able to pass the blood–brain barrier, might contribute to preventing nerve cell dysfunctions and BDNF downregulation that lead to neurodegeneration both in vitro and in vivo [57,58,59]. In neurodegenerative disorders, polyphenols protect neuronal cells by neurotrophic factor-mimic activity, in addition to suppression of apoptosis signaling in the mitochondria and modulation of gene expression (i.e., genes coding anti-apoptotic Bcl-2 and neurotrophic factors, such as brain-derived and the glial cell-line-derived neurotrophic factor) (for review see [60]). In the current study, semi-quantitative analysis of neurodegeneration revealed no significant effect of ethanol and/or stress in the CeA when the rats are chronically treated with AO. Notably, we demonstrated that AO treatment during ethanol drinking significantly reduced CeA neuronal loss in the AO-treated IEI/UMS rats compared to the water-treated IEI/UMS ones.

Recently, studies in 158 N and BV-2 nerve cells show that AO from Agadir and Berkane (the two main producing regions of AO in Morocco) is able to attenuate the cytotoxic effects of 7-ketocholesterol (7KC), which is increased in the brain of patients with Alzheimer’s disease: loss of cell adhesion, cell growth inhibition, increased plasma membrane permeability, attenuation of reactive oxygen species (ROS) overproduction, mitochondrial, peroxisomal and lysosomal dysfunction, and a decrease in mRNA levels of the nuclear peroxisome proliferator-activated receptor α (PPARα) [61,62]. Neurodegeneration is also characterized by increased oxidative stress which can contribute to lipid peroxidation, protein carbonylation, damage to organelles (mitochondria, lysosomes, and peroxisomes), and DNA alterations, which can induce numerous dysfunctions in nerve cells and ultimately lead to cell death [63,64,65]. Our study also claims that ethanol-stress-induced alcohol binge-like drinking drastically altered the amygdala pro-oxidant/antioxidant balance, as indicated by the increased MDA and NO content versus the decrease in CAT and SOD enzymatic activities. However, AO supplementation upregulated these antioxidant enzyme levels and was found to be an efficient suppressor of oxidative stress under ethanol-stress exposure. These results, which extend our previous findings, show that AO supplementation, by reducing voluntary ethanol consumption and ethanol preference in rats, reduced oxidative stress and neurodegeneration in the brain after adolescent IEI exposure [19]. Additionally, we found that AO pre-treatment improved memory function in adolescent IEI-exposure rats and restricted stress oxidation in the hippocampus and PFC [29]. Neuronal loss recovery and amygdala oxidative stress upregulation by AO supplementation has been also found in rats exposed to UMS [20]. Accordingly, in a pilocarpine model of induced status epilepticus, Bahbiti and co-workers have shown that AO pretreatment at the dose of 10 mL/kg/day attenuates oxidative stress in the hippocampus of rats [66].

In summary, AO supplementation reduces voluntary ethanol consumption, alleviates oxidative stress, and minimizes neurodegeneration in the amygdala, offering protective effects against the long-lasting consequences of adolescent ethanol-stress exposure.

## 4. Materials and Methods

### 4.1. Ethics Statement and Animal Subjects

All experimental procedures were in compliance with the National Institute of Health (NIH) Guide for Care and Use of Laboratory Animals and approved by the Center for Doctoral Studies of the Faculty of Science at Ibn Tofail University (Kenitra, Morocco). During treatment, behavioral testing and tissue collection procedures were devised to minimize the potential pain and distress of the animals used in this study. All rats were frequently monitored, at least 3 times a week, for health status.

Male Wistar rats born in our research animal facility were weaned at PND 21 and housed under a 12 h light/12 h dark cycle (lights on between 07:00 a.m. and 7:00 p.m.) in a humidity- and temperature-controlled room (21 ± 1 °C and 55–60%, respectively). The rats were provided with ad-libitum access to a standard rodent diet and tap water, except when one of these parameters was changed for the alcohol drinking regime. For the following experiments, animals remained housed five per cage except for the ethanol consumption experiments, for which rats were individually housed. The rats were randomly allocated to water-treated and AO-treated cohorts (without/with orally AO treatment, respectively), and each cohort was divided into the following treatment onset conditions:

- Control group (Ctrl): untreated or supplemented with AO (*n* = 17);
- Ethanol intoxicated group (IEI): submitted to adolescent IEI with/without AO treatment; (*n* = 18)
- Stressed group (UMS): submitted to adolescent UMS with/without AO treatment (*n* = 17);
- Ethanol intoxicated and stressed group (IEI/UMS): submitted to adolescent IEI and UMS with/without AO treatment (*n* = 17).

**Figure 4:**
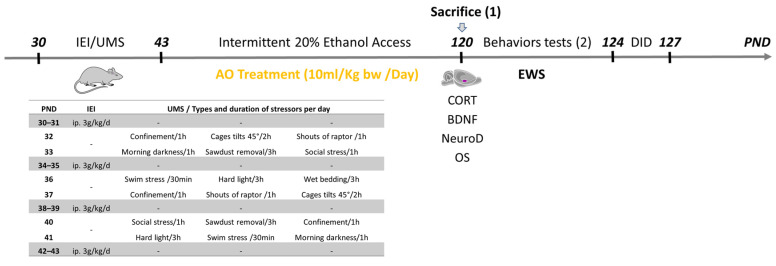
Experimental timeline and daily schedules for the intermittent ethanol intoxications (IEI) and the unpredictable mild stress (UMS). From post-natal day (PND) 30 to PND 43, IEI animals received a single daily intraperitoneal (ip) administration of ethanol (3 g/kg, 20% ethanol *w*/*v*) in the AM on a 2-days-on/2-days-off schedule and Ctrl subjects received comparable volumes of 0.9% saline. On alternate days, the rats were submitted to UMS protocol. The home-cage voluntary intermittent 20% ethanol consumption was measured on a two-bottle choice procedure (from PND 44 to PND 120). During the same period, the rats were treated daily by intragastric gavage with Argan oil (AO, 15 mL/kg–bw). Next, plasma corticosterone (CORT) levels were measured from tail bloods samples in all rats during the last day of AO treatment (between 6:00 p.m. and 7:00 p.m.). One day later, separate groups of each experimentally conditioned rats were used to measure ethanol withdrawal signs (EWS) and binge-like drinking (on the drinking in the dark paradigm (DID)) (2), or were euthanized for histochemical and the biochemical analyses, to measure the neurodegeneration (NeuroD) levels, the brain-derived neurotrophic factor (BDNF) levels and the oxidative stress (OS) in the amygdala (1).

Some rats (five rats per group), were used only for histological and biochemical evaluations, immediately after the AO treatment period. These rats never underwent any behavioral tests. Figure 4, illustrates the treatment and behavioral timeline for rats undergoing adolescent IEI/UMS; the treatment conditions are defined in detail below.

### 4.2. Adolescent IEI Procedure

For the adolescent IEI or normal saline (Ctrl) exposure, adolescent rats received intraperitoneal (i.p.) injections of either ethanol (3 g/kg, 20% *w*/*v*) or the equivalent volume of 0.9% (*w*/*v*) saline during PND 30–43 on a 2-days-on and 2-days-off basis. This paradigm of ethanol exposure has been used by other investigators as well [67,68,69]. Specifically, the pups were injected at PND 30–31, 34–35, 38–39 and 42–43. In this way and for 2 weeks, each young rat received eight alcohol doses simulating the “binge drinking” pattern, with withdrawal-like periods (at PND 32–33, 36–37 and 40–41).

### 4.3. Adolescent UMS Procedure

The UMS procedure was performed as described by [70] with minor modifications. On alternate days of the adolescent IEI procedure (during sessions with no ethanol injections, at PND 32–33, 36–37, and 40–41), the rats (UMS and IEI/UMS subjects) were submitted to unpredictable stressing events three times per 24 h. The stress regimen consisted of a 1 h of period of confinement in a restriction tube (20 cm long, 8 cm diameter) with unexpected noises, 30 min of forced bath in 30 °C water, 3 h of paired housing in damp sawdust, 2 h of cage tilts at 45°, 1 h of social stress by changing partners in the cage, 3 h of sawdust removal, 1 h of darkness in the morning, 1 h of hard light, or 30 min of shouts of a raptor. The sequence and moment of stressing events were randomly organized to avoid habituation or foresight and maximize their stressing effects. An interval of 1 h between the three stressor events per day was respected. For ethical reasons, all nociceptive stressors were excluded as well as water and food deprivation. Ctrl and IEI groups rats were housed in separate rooms and had no contact with the stressed animals; they were kept undisturbed with the exception of during necessary procedures such as cage cleaning and ethanol or saline injections. The daily schedule for the UMS is summarized in Figure 4.

### 4.4. AO Treatment Procedure

On PND 44, 1 day after the adolescent ethanol binge-like exposure, the rats (cohort 2) were treated daily by AO intragastric gavage (15 ml of AO/kg–bw, between 9:00 a.m. and 10:00 a.m.) for 11 weeks. Concurrently, cohort 1 animals (water-treated rats) were administered orally with distilled water following the same schedule. At the same time, for 3 months, body weights were measured weekly. The current procedure of AO treatment was based on previous studies from our group [19,29,71] showing that AO supplementation (10 mL/kg/day) during the developing central nervous system (CNS) may induce neuroprotective effect against IEI or UMS-induced long-lasting neurobehavioral impairments and brain histochemical alterations in rats. The AO used in this study was obtained from the argan fruit of *Argania spinosa* L., an endemic tree located mainly in south-western Morocco [21]. It has a composition similar to that used in human food [72,73] and was extracted from fresh seeds by artisanal methods without any preliminary treatment. It was preserved at room temperature in a bottle made of brown glass.

### 4.5. Ethanol Intake in Two-Bottle Intermittent Free Choice Test

To assess alcohol voluntary consumption and preference after the adolescent IEI and/or UMS exposure, the animals were single-housed (on PND44) and trained to drink in a two-bottle choice procedure. In total, the experiment lasted for 11 weeks, during the same period of AO treatment. First, rats were given 1 week to habituate to a two-bottle free choice situation and to alcohol self-administration. During this period, rats were allowed access to two bottles, one containing plain tap water and the other containing a sweetened ethanol (with 1% sucrose). The concentration of ethanol was raised every day, increasing from 2 to 20% (*v*/*v*). Then, the rats had intermittent access to a bottle of unsweetened 20% ethanol and a bottle of tap water for 24 h every other day, during the following 10 weeks (i.e., from PND52 to PND121). The consumption of ethanol and water was measured daily (between 8:00 am and 11:00 am), to calculate both the ethanol intake (g/kg/day) and the preference ratio (ml ethanol/mL total fluid intake). The density of alcohol (0.7894 g/L) was taken into account in the calculations. The positions of the bottles were changed at the same time as the fluid consumption measurements.

### 4.6. Behavioral Testing

At the end of the intermittent 20% ethanol access period, the AO treatment was stopped and ethanol withdrawal signs (EWS) and binge-like drinking were evaluated in five or six subjects for each experimental group. The drinking in the dark (DID) procedure was used to assess the binge-like drinking (relapse risk). Separate representative rats were immediately euthanized and destined for the biochemical (five per group) and histochemical (five per group) evaluations.

EWS. Overt EWS was assessed (for five or six rats per group) 1 day following the intermittent 20% ethanol consumption (on PND 121 at 9:00 a.m.). Using a withdrawal rating scale adapted from [74], ethanol withdrawal signs, including agitation (irritability to touch), tail stiffness, abnormal posture, gait, and stereotyped signs (grooming, sniffing, head weaving, gnawing, and chewing) were scored at different time points: at 0 (before the removal of alcohol), 2, 6, 24, 48, and 72 h post-ethanol withdrawal (between PND 121 and PND 124). Each sign was assigned a score of 0 to 5 and quantified for 10 min by two blind observers to the treatment in the animal’s home cages. The global EWS score (sum of the five observation scores; 0 to 25) was used to quantify withdrawal severity.

DID. The relapse-like drinking during ethanol withdrawal was assessed using the modified-DID procedure as adapted from [75]. Briefly, the rats were allowed a restricted free choice to a two-bottle at 1 h into the dark cycle during 4 consecutive days after EWS evaluation (i.e., from PND 124 to PND 127). The test consisted of a 2 h of access to two bottles, one containing 20% (*v*/*v*) ethanol and the other containing tap water. The concentration of ethanol was raised every day, increasing from 5% on the first day to 20% on the fourth day. The amount of alcohol consumed (volume) was recorded immediately after each drinking session and converted to grams per kilogram using each animal’s alcohol consumption and body weight. On the fourth day, average ethanol consumption/2 h was used as an index of relapse-like drinking.

### 4.7. Histochemical Evaluation

#### 4.7.1. Perfusion, Brain Isolation and Histology

Immediately at the end of AO treatment and after 11 weeks of intermittent 20% ethanol consumption (on PND 121), five representative rats/group were deeply anesthetized with chloral hydrate 7% (5 mL/kg i.p.) and transcranially perfused with heparinized saline (0.9% sodium chloride, 1000 UI heparin) for 2 min, followed by 4% paraformaldehyde in 0.1 M phosphate buffered saline (PBS, pH 7.4). The brains were collected, weighed, and post-fixed for 12 h in the same fixative, and cryoprotected in 20% sucrose/0.1 M PBS at 4 °C over 24 h. The following day (PND 122), 30 μm serial slices were made with vibratome (VT1000 S, LEIKA Biosystems, Nussloch, CA, USA) and maintained at 4 °C in a glycerol solution. Every section was mounted on gelatin-coated slides and dried overnight. Staining was performed with Cresyl violet for histological examination as previously described [76]. The amygdala, in particular the CeA neurons, were visualized under an optical microscope with a professional HDMI camera (B-500TiFL, OPTIKA S.r.l., Ponteranica, Italy). To highlight any differences in the number and size of morphological elements, slices were observed at several magnifications (10×, 20× and 40×).

#### 4.7.2. Neurodegeneration in the CeA

Neurodegeneration evaluation was performed in Nissl-stained coronal brain sections in the CeA, according to the method described in our previous studies [19]. Using a grading system as previously described [77], the severity of neuronal damage was semi-quantitatively assessed: score 0, no obvious damage; score 1, slightly abnormal appearance of the structure without clear evidence of visible neuronal loss; score 2, lesions involving 20–50% of neurons; and score 3, lesions involving >50% of neurons. Scoring was performed in the CeA brain subregion in four sections per rat [30]. Furthermore, the amount of polymorph neurons in this brain subregion was quantified as described earlier [78] using ImageJ 1.45 software (Microsoft Java, Redmond, WA, USA). Only cells of neuronal morphology and a diameter larger than 8 µm were counted. For each rat, three sections were analyzed, and the numbers of neurons were averaged from each structure section. Average scores from these sections of both hemispheres in each rat were used for the calculation of group data.

#### 4.7.3. BDNF Immunohistochemistry in the CeA

Some sections (30 µm) from amygdala subregion, obtained from the previous five representative rats/group, were submitted to immunohistochemistry analysis. In order to detect the BDNF protein in the CeA, we ran free-floating sections under moderate shaking using the avidin–biotin system method (Vectastain ABC System), as previously described [79]. Sections were rinsed in Tris-buffered saline and then incubated in H_2_O_2_ and 10% methanol to block endogenous peroxidase activity followed by 48 h incubation at 4 °C with anti-BDNF (1:1000) diluted in 0.01 M PBS (pH 7.4), containing 0.5% Triton X-100 and normal goat serum. For the avidin–biotin system, sections were incubated with the appropriate biotinylated secondary antibodies diluted at 1/250 in PBS for 1 h followed by the avidin–biotin–peroxidase complex (diluted 1:200; Immuno Pure ABC peroxidase staining kit) and developed in a solution of 0.015% 3,3-diaminobenzidine, 0.0024% H_2_O_2_ in 0.05 M Tris–HCl (pH 7.6). After adhesion on gelatin-coated slides, sections were dehydrated and cover slipped with Histo-kitt. Illustrative images of the CeA from all experimental groups were obtained with a professional HDMI camera attached to a microscope (B-500TiFL, OPTIKA S.r.l., Ponteranica, Italy). Images were later analyzed using ImageJ software by researchers blinded to the experimental conditions of the representative animals. A 250 µm^2^ reticule was placed over the region of interest from the antero-posterior −1.88 mm to −3.80 mm from Bregma according to the atlas of Paxinos and Watson (2013) [30]. All BDNF-positive cells were counted within the reticule for three corresponding bilateral sections (1.0 mm) for representative animals (*n* = five) in each experimental group. The cell density was expressed as the number of BDNF-immunoreactive (IR) somata within the 250 µm^2^ reticule.

#### 4.7.4. Corticosterone Blood Levels and Oxidative Stress in the Amygdala

On PND 121, plasma CORT levels were measured from tail bloods samples in all rats during the last day of AO treatment (between 6:00 p.m. and 7:00 p.m.). The blood sample was put into a dry tube for plasma extraction and analysis of corticosterone blood levels (ng/mL) was measured by chemiluminescence (CMIA) on an automaton architect system Abbott, using a commercial kit. Subsequently, the brains from five separate representative rats were quickly removed and cooled on dry ice. Then, the amygdala was dissected on ice using a rat brain atlas for reference (Bregma −2.12 to −4.30). The brain tissues were homogenized in ice-cold 20-mM Tris-HCl buffer (pH 7.4) for the determination of the lipid peroxidation level (LPO), nitrite content (NO), catalase (CAT), and superoxide dismutase (SOD) activities.

Oxidative stress parameters in the amygdala were evaluated as previously described in our recent study [19] using spectrophotometric methods.

Briefly, NO assay was performed using the Griess reagent (0.1% N-(1-naphthyl) ethylene diamine dihydrochloride; 1% sulfanilamide in 5% phosphoric acid; 1:1), and the results were expressed as μmol of nitrite content/g of homogenate.

LPO assay was evaluated by measuring the thiobarbituric-acid-reacting substances in homogenates, and the results were expressed as nmol of malondialdehyde (MDA)/g wet tissue.

CAT activity was measured by the method that uses H_2_O_2_ to generate H_2_O and O_2_. The results are expressed as mmol/min/mg of protein.

SOD activity was evaluated by measuring its ability to inhibit the photoreduction of nitroblue tetrazolium. The results are expressed as mmol/min/mg of protein.

### 4.8. Statistical Analysis

Results are expressed as the mean ± the standard error of the mean (SEM). Statistical comparisons were conducted using three-way analysis of variance (ANOVA) followed by a Tukey post-hoc multiple comparison test. Nonparametric data resulting from semi-quantitative analysis of histological staining were analyzed by Kruskal–Wallis ANOVA, followed by a Dunn’s post-hoc comparison test. Differences were considered statistically significant if the *p*-value was less than 0.05. Data analysis was performed with a Graph Pad Prism 8.00 (Graph Pad Software Inc., La Jolla, CA, USA).

## 5. Conclusions

The present study demonstrates that combined adolescent binge-like ethanol exposure and mild stress in rats exacerbate ethanol consumption, withdrawal signs, and relapse-like drinking in adulthood. This co-exposure also led to significant neurobiological alterations, including increased plasma CORT levels, oxidative stress, neurodegeneration, and reduced BDNF expression in the CeA. Notably, these detrimental effects were most pronounced in rats subjected to both IEI and UMS, with UMS alone showing no significant impact. These findings align with previous studies suggesting that the interaction between early alcohol exposure and stress amplifies the negative consequences of each factor for brain function and behavior.

**Figure 5:**
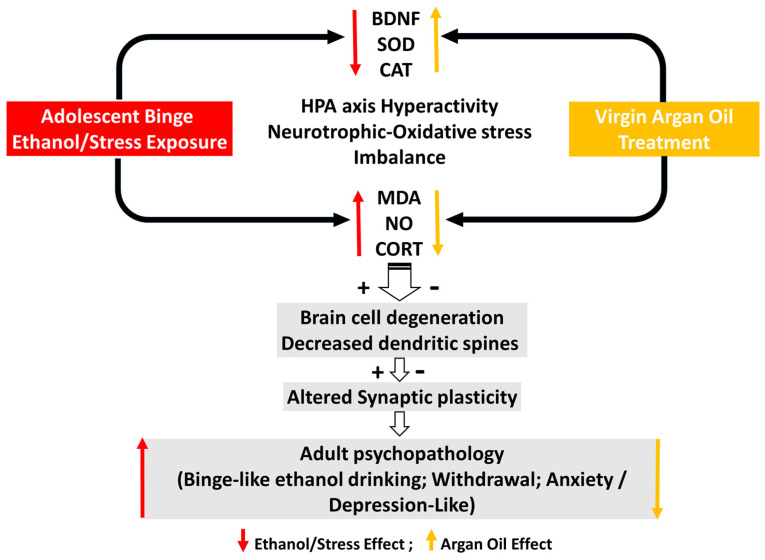
Possible mechanisms proposed for the “therapeutic” effects of AO against adolescent binge-like/mild stress exposure. Our study suggests that virgin AO supplementation during voluntary ethanol consumption, exerted addictolytic-like effects in IEI/UMS rats, mediated in part by normalizing plasma CORT levels, reducing brain neuronal loss while upregulating BDNF levels and oxidative stress imbalance in the amygdala.

On the other hand, the administration of AO at a dose of 15 mL/kg/day during voluntary 20% ethanol access provided substantial protective effects against these adverse outcomes. As summarized in Figure 5, AO treatment attenuated withdrawal signs, normalized plasma CORT and BDNF levels, and reduced oxidative stress and neurodegeneration in the CeA. Although AO did not significantly affect voluntary ethanol consumption in the IEI/UMS group, it reduced relapse-like drinking after ethanol deprivation. Knowing the interesting chemical composition of AO and the complexity of AUD pathophysiology, we can consider the possible synergistic effects of these phytochemical compounds that would be more beneficial than the use of each one.

In sum, this study highlights the long-lasting neurobiological and behavioral consequences of adolescent alcohol-stress co-exposure, while underscoring the potential therapeutic value of AO in reducing ethanol-related harm. These findings support the development of AO-based interventions for managing AUD, particularly in individuals with a history of stress exposure. The results provide clear evidence for the “addictolytic properties” of AO, highlighting the need for further research into its neuroprotective mechanisms and potential clinical applications.

## Figures and Tables

**Table 1:** Corticosterone levels in the plasma and oxidative stress in the amygdala.

Conditions		Water				AO			
		Ctrl	IEI	UMS	IEI/UMS	Ctrl	IEI	UMS	IEI/UMS
CORT in plasma (ng/mL)		90.34 ± 14.89	140.21 ± 9.56 ^a^	105.83 ± 11.09	147.23 ± 5.74 ^aa^	84.34 ± 12.11	108.81 ± 6.27	106.23 ± 9.77	127.72 ± 10.07
OS in Amygdala	MDA	5.24 ± 0.67	11.34 ± 1.81 ^a^	6.59 ± 0.95	12.34 ± 1.19 ^aac^	4.43 ± 0.80	8.74 ± 1.54	5.39 ± 0.60	7.34 ± 0.94 ^δ^
	NO	73.07 ± 12.09	119.09 ± 9.74 ^a^	86.32 ± 7.59	125.89 ± 9.58 ^aa^	78.67 ± 13.03	101.09 ± 6.86	76.32 ± 7.59	109.69 ± 8.35
	SOD	67.09 ± 9.64	31.19 ± 6.67 ^a^	54.06 ± 5.58	21.39 ± 5.45 ^aa^	65.29 ± 8.37	55.19 ± 8.19	60.06 ± 10.27	58.19 ± 6.26 ^δ^
	CAT	7.41 ± 0.85	4.12 ± 0.99	6.62 ± 1.04	2.72 ± 0.61 ^a^	8.01 ± 1.24	5.92 ± 0.93	7.22 ± 1.02	6.12 ± 0.67

Plasma corticosterone (CORT) levels were measured from tail bloods samples in all rats during the last day of AO treatment (between 6:00 p.m. and 7:00 p.m.). Oxidative stress (OS) was measured in the amygdala brain subregion, 24 h after 11 weeks of intermittent 20% *v*/*v* ethanol free access. The results are expressed as mean ± SEM (from 5 representative rats per group) of the MDA: Malondialdehyde (nmol/g of homogenate); NO: Nitrite content (μmol/g of homogenate); SOD: Superoxyde dismutase activity (µmol/min/mg of protein) and CAT: Catalase activity (µmol/min/mg of protein) in Ctrl, IEI, UMS and IEI/UMS rats with or without AO treatment. ^a^ *p* < 0.05, ^aa^ *p* < 0.01 compared with Ctrl; ^c^ *p* < 0.05 compared with UMS; ^δ^ *p* < 0.05 AO effect, according to three-way ANOVA, followed by Tukey’s test.

## Data Availability

All data are contained in this article and there are no repository data.
